# General practitioners’ views on the acceptability and applicability of a web-based intervention to reduce antibiotic prescribing for acute cough in multiple European countries: a qualitative study prior to a randomised trial

**DOI:** 10.1186/1471-2296-13-101

**Published:** 2012-10-11

**Authors:** Sibyl Anthierens, Sarah Tonkin-Crine, Elaine Douglas, Patricia Fernandez-Vandellos, Jaroslaw Krawczyk, Carl Llor, Jochen WL Cals, Nick A Francis, Lucy Yardley, Samuel Coenen, Theo Verheij, Herman Goossens, Paul Little

**Affiliations:** 1 Centre for General Practice, Vaccine & Infectious Disease Institute (VAXINFECTIO), Laboratory of microbiology, University of Antwerp, Antwerp, Belgium; 2 Primary Care and Population Sciences, Faculty of Medicine, University of Southampton, Southampton, SO16 5ST, UK; 3 Department of Epidemiology & Public Health, University College London, London, UK; 4 Applied Research in Respiratory Diseases, Hospital Clinic of Barcelona, Barcelona, Spain; 5 Department of Family and Community Medicine, Medical University of Lodz, Lodz, Poland; 6 Primary Care Centre Jaume I, University Rovira i Virgili, Tarragona, Spain; 7 Department of General Practice, CAPHRI School for Public Health and Primary Care, Maastricht University, Maastricht, the Netherlands; 8 Cochrane Institute of Primary Care and Public Health, School of Medicine, Cardiff University, Cardiff, UK; 9 Faculty of Human and Social Sciences, University of Southampton, Southampton, UK; 10 Julius Centre for Health Sciences and Primary Care, University Medical Center Utrecht, Utrecht, Netherlands; 11 Laboratory of Microbiology, Vaccine & Infectious Disease Institute (VAXINFECTIO), University of Antwerp, Antwerp, Belgium

**Keywords:** Primary health care, Behaviour change intervention, Implementation, Attitude of health personnel, Qualitative research

## Abstract

**Background:**

Interventions to promote prudent antibiotic prescribing by general practitioners (GPs) have often only been developed for use in one country. We aimed to develop an intervention which would be appropriate to implement in multiple European countries in order to offer greater benefit to practice whilst using fewer resources. The INTRO (INternet TRaining for antibiOtic use) intervention needed to deliver training to GPs in the use of C-Reactive Protein (CRP) near patient tests to help diagnose acute cough and in communication skills to help explain prescribing decisions to patients. We explored GPs’ views on the initial version of INTRO to test acceptability and potentially increase applicability for use in multiple countries before the start of a randomised trial.

**Method:**

30 GPs from five countries (Belgium, England, the Netherlands, Poland and Spain), were interviewed using a “think aloud” approach. GPs were asked to work through the intervention and discuss their views on the content and format in relation to following the intervention in their own practice. GPs viewed the same intervention but versions were created in five languages. Data were coded using thematic analysis.

**Results:**

GPs in all five countries reported the view that the intervention addressed an important topic, was broadly acceptable and feasible to use, and would be a useful tool to help improve clinical practice. However, GPs in the different countries identified aspects of the intervention that did not reflect their national culture or healthcare system. These included perceived differences in communication style used in the consultation, consultation length and the stage of illness at which patient typically presented.

**Conclusion:**

An online intervention to support evidence-based use of antibiotics is acceptable and feasible to implement amongst GPs in multiple countries. However, tailoring of the intervention to suit national contexts was necessary by adding local information and placing more emphasis on the fact that GPs could select the communication skills they wished to use in practice. Using think aloud methods to complement the development of interventions is a powerful method to identify regional contextual barriers to intervention implementation.

## Background

Whilst primary care guidelines promote prudent antibiotic use, prescribing of antibiotics in primary care across European countries continues to be high [1], with studies indicating that unnecessary prescribing is still common in even countries with the lowest prescribing [2]. Many types of interventions to change GPs’ behaviours regarding antibiotic prescribing, for mostly self-limiting respiratory infections, have been developed [3-5]. However, most have been designed for implementation in a single context [6-8] and are therefore focused on specific countries, health care organisations and cultures. As a result, the effectiveness of an intervention in alternative contexts cannot be established. Exploring whether an intervention that is effective in one country can also be effective in others may provide opportunities for changing GP behaviour on a wider scale whilst using fewer resources. Tonkin-Crine et al. found consistency amongst views of GPs from a number of countries in terms of components that should be included in an intervention aimed at improving antibiotic prescribing for common infections, and subsequently concluded that a single intervention could be developed that would be viewed as acceptable and feasible by GPs in a range of countries [9].

Previous research has supported the use of qualitative investigations alongside trials to help explain the effects of interventions [10] but also to improve their design before running a trial [11,12]. For successful uptake and implementation it is important that the content, design and delivery of interventions are viewed as acceptable by the target audience in different countries. In order to prepare a novel intervention for use in multiple European countries it is important to assess the perceptions and expectations of the subjects of the intervention. Delivering an intervention, in which both content and format are viewed positively by GPs, may result in higher uptake, greater adherence and, subsequently, greater change in behaviour. It is also important to understand whether an intervention is feasible to be implemented in the context that it is intended to be used.

The current study explores GPs’ views of a web-based intervention to assess the acceptability of the intervention across countries and to increase the applicability of the final version, resulting in an acceptable and feasible version for implementation in a randomised trial in multiple countries. We also aimed to see whether, when presented with a “real-life” intervention, ready for implementation, GPs’ views between countries remain similar or whether contextual differences appear. The actual trial of the intervention only commenced after this qualitative work had finished. Trial results will be reported once patient recruitment and analysis have finished.

## Method

### Developing the intervention

The INTRO (INternet TRaining for antibiOtic use) intervention was designed as part of a European study, GRACE (Genomics to combat Resistance against Antibiotics in Community-acquired LRTI in Europe), as a programme to reduce inappropriate antibiotic prescribing for lower respiratory tract infections (LRTIs) in European primary care. The initial version of INTRO was developed by a collaboration of European multi-disciplinary researchers, representing six countries of interest (Belgium, England, the Netherlands, Poland, Spain and Wales). The INTRO intervention was originally written in English and then translated to produce five versions with identical content but in different languages (Flemish, English, Dutch, Polish and Spanish). Both the content and format of the intervention were a result of continuous discussion by all members of the team. The content of INTRO is based on previous research indicating the effectiveness of interventions which contain communication skills training for GPs, supported by a patient booklet, and/or the use of a C-reactive protein (CRP) near patient test. Such interventions have been found to significantly reduce antibiotic prescription rates in patients with LRTIs [5-7,13-15].

**Figure 1:**
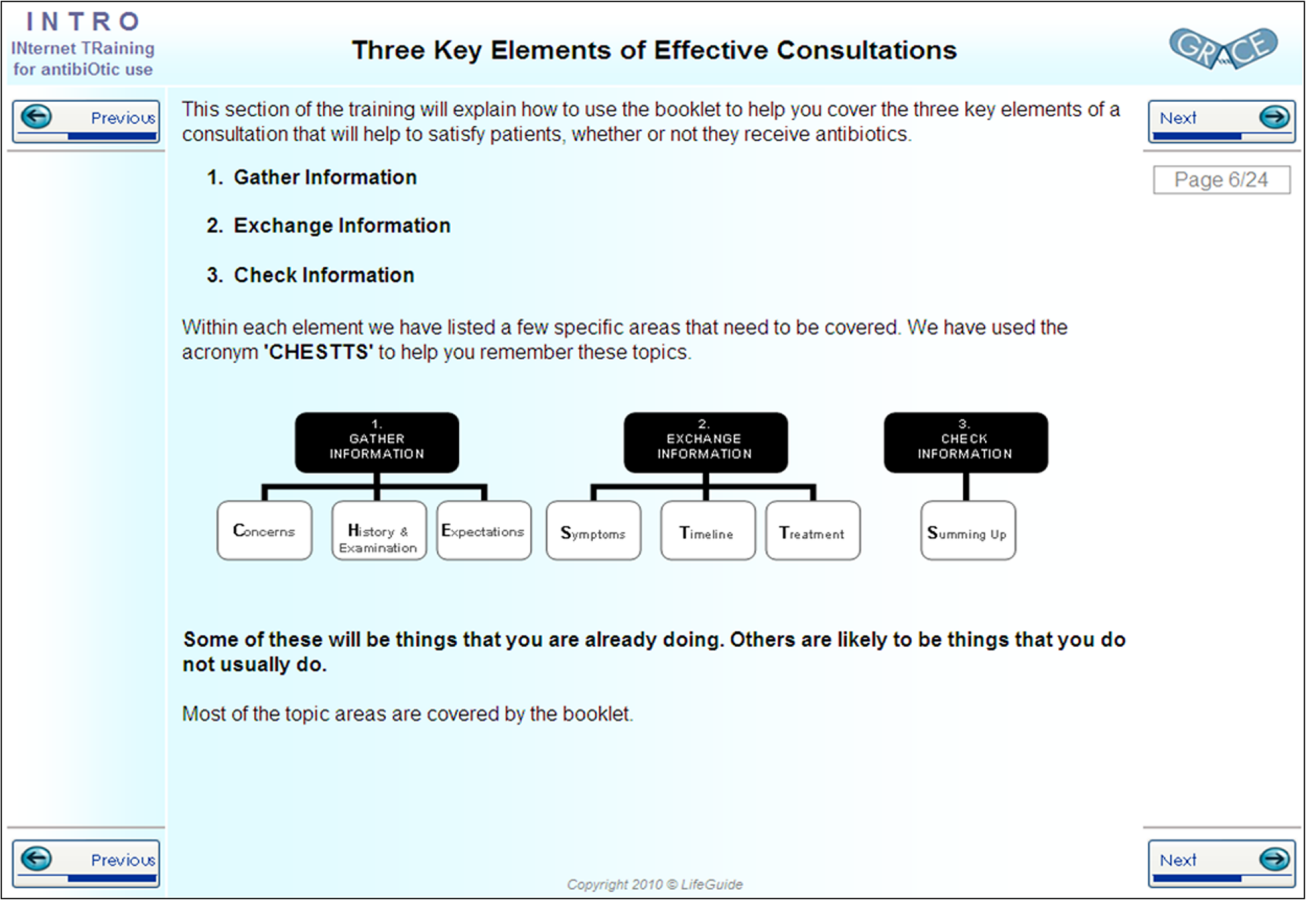
One web-page from the communication skills training included in the INTRO intervention.

INTRO has three main sections; an introduction, communication skills training and CRP training. The introduction gives information about the need for reduced antibiotic prescribing and the effect of over prescription on the health care system, patients and GPs. The communication training provides videos showing how a GP can use communication skills to; elicit patient concerns and expectations, discuss the pros and cons of antibiotic treatment and the natural course of LRTIs, whilst maintaining patient satisfaction. The communication training is supported by a patient booklet to be discussed in the consultation. The CRP training introduces the CRP test as a method to assist diagnosis of acute cough, trains GPs in the use of the test and gives instructions on its use within a consultation. The training is delivered in a self-directed web-based format. Figure 1 shows an example of one webpage in the communication training.

### Participants

Ethical approval for the whole of the GRACE INTRO project was granted by Southampton and South West Hampshire Local Research Ethics Committee (ref. 10/H0502/29). The four research sites outside of the UK also obtained ethical approval from their local organisations; the Committee for Medical Ethics (Comité voor medische ethiek), University Hospital Antwerp, Belgium; the Medical Ethics Committee (Medisch Ethische Toetsing Commissie, METC), Utrecht, the Netherlands; Barcelona Clinic Ethics Committee of Clinical Research (Hospital de Barcelona y secretaria del Comité Ėtico Investigación Clinica, CEIC) and the Primary Care Research Institute (Institut d’Investigació en Atenció Primaria IDIAP Jordi Gol), Barcelona, Spain and the Bioethics Committee of The Medical University of Lodz (Komisja Bioetyki Universytetu Medycznego w Lodzi), Poland. GPs were recruited from five countries, Belgium, England, the Netherlands, Poland and Spain. We used convenience and purposeful sampling to recruit GPs with a range of demographics and primary care experience. GPs were identified from local practices by researchers in each country and were invited to participate in the study by email or phone. GPs were contacted up to three times if a response was not received. We were not able to collect information on those GPs who did not respond or their reasons for not responding. We initially aimed to recruit 25-30 GPs in total and recruitment continued until no new themes appeared to be emerging from the data.

### Interviews

Five experienced primary care researchers completed interviews across the five countries. All interviewers participated in a teleconference, led by an experienced qualitative researcher, prior to interviews in order to standardise the interview approach. GPs were interviewed face to face in their own practice. GPs were sent information about the study prior to interviews and were given a chance to read the consent form. All GPs gave written consent to take part in the study and researchers informed GPs that all interview data would remain confidential and anonymous. Interviews used a “think aloud” method [16,17]. Unlike traditional qualitative interviewing, the ‘think aloud’ approach focuses on respondents verbalising their initial thoughts whilst viewing intervention materials. This approach allowed interviewers to follow GPs’ thought processes and to identify any problems with comprehension and usability of the intervention. Questions were asked to clarify the meaning of statements or to prompt the user to continue to comment should they lapse into silence. Once each GP had completed the “think aloud” section of the interview some semi-structured interview questions were asked about the intervention as a whole. This part of the interview allowed us to explore views on the acceptability and applicability of the web-based intervention in the local setting of the individual GP as well as routine national primary care practice. Interviews were audio recorded and transcribed verbatim. Interviews undertaken in countries other than the UK were translated into English by translators in each country and translations were checked by the original interviewer.

### Data analysis

Interviews were analysed by two researchers (SA and STC) in the UK and Belgium using thematic analysis [18]. Thematic analysis allows an inductive approach to ensure that emerging themes are grounded in the original data and that influence from researchers’ preconceptions is reduced. Transcripts were coded line by line to produce initial codes; these were then compared for similarities and differences to begin forming themes. 14 transcripts (at least two from each country) were coded independently by SA and STC and then the two sets of themes produced by each researcher were compared and discussed. Differences or disagreement between themes were discussed by SA, STC and ED and consensus decisions were reached to produce an agreed set of themes. SA used these themes to analyse the remaining 16 interview transcripts. Any data which did not fit into the themes were discussed between the researchers and new themes and subthemes were added where necessary. Analysis used a constant comparative approach [19] where researchers moved back and forth between the data and emerging themes until all data had been analysed. NVivo 8 was used to facilitate coding.

## Results

### Participant characteristics

30 GPs were interviewed between June and September 2010, 6 from each of the five countries. Interviews lasted between 35-120 minutes, with no major differences in interview lengths between countries. GPs from the UK were on average slightly younger than those from other countries and GPs from Belgium had, on average, more years in practice than colleagues in other countries (Table 1).

**Table 1:** Demographics of 30 GPs interviewed in the five countries

	**Belgium (N = 6)**	**UK (N = 6)**	**Poland (N = 6)**	**Spain (N = 6)**	**Netherlands (N = 6)**
**Mean age (yrs)**	40.2	33.3	37.5	35.8	41.3
**Age range (yrs)**	29-51	29-36	32-48	26-45	31-49
**Mean practice experience (yrs)**	17.1	4.4	6	9	10
**Male**	2	4	2	1	3

### Findings

Four major themes emerged, and these were relevant to GPs from all five countries. We specifically chose to report only two of the four themes in this paper as they contain instances where GPs’ views indicated differences based on country-specific context. The two themes which are not discussed here cover aspects related to practical aspects of the web-based format of the INTRO intervention. Theme 3, “Ease of use of the intervention format” included GPs’ comments about the attractiveness of the website and how easy it was to navigate. In response to these comments we made changes to the website such as adding a progress bar so GPs were aware of how much of the training programme they had worked through at any one point. Theme 4, “Clarity of the intervention content”, included GPs’ comments on points in the website where there was confusion. These included cross-country examples such as a graph being difficult to understand or web pages containing text which was “too scientific”. It also included country specific examples. GPs from countries outside the UK disliked having to read subtitles when videos had been filmed in English. GPs from Belgium, Spain and Poland also initially misunderstood CRP test results because they were used to working with different measurements (mg/dl rather than mg/l). Comments within these two themes were fed back to assist the development of the final version of INTRO. Major changes to the website included simplifying the main menu and adding pages to indicate when GPs had completed one of the training sessions. Other changes included adding measurements (mg/dl) to the CRP materials alongside mg/l figures for materials to be used in Spain, Poland and Belgium and also removing self-care advice regarding Echinacea, Vitamin C and cough medicines from the Dutch patient booklets as the information was not in line with national guidelines.

All four themes are listed in Table 2, with the themes that are discussed here highlighted in bold.

**Table 2:** Emerging themes from interviews with GPs asking about views on the INTRO intervention

**Relevance of the intervention: awareness of the problem of antibiotic prescribing**	
· Relevant topic but difficult to tackle	
· GP’s existing knowledge affects usefulness of intervention	
**Attractiveness and feasibility of the intervention**	
· Making the intervention worthwhile for GPs	
· Considering the needs of patients	
· Managing patient demand	
· Trade-off between benefit and harm to consultation time	
· Feasibility in the primary care context	
· Relevance to the national context and established local practices	
· Providing evidence based information	
Ease of use of the intervention format	
· Design to make easy to use	
· Employ attention grabbing features	
Clarity of the intervention content	
· Make content simple to understand	
· Content should be country relevant	
· Use content to guide GP through intervention components	

#### Relevance of the intervention: Awareness of the problem of antibiotic prescribing

GPs in each country agreed with the importance of the intervention’s aim to promote prudent antibiotic use. In addition, they identified with the experience of having difficulties in prudent prescribing for LRTI in their own practice.

"“It is useful to do this study because it just reminds you why you should continue doing what you are doing, because I think after a while GPs forget about antibiotic resistance and how dangerous it is to the patient.” (British, GP2)."

GPs from Poland appeared to particularly like the intervention as they reported having little prior education on this topic.

"“I think that the topic is very interesting. The training is completely new for us. I think that we over-prescribe in Poland. This is a completely new approach for me and I find it very interesting.” (Polish, GP1)."

In contrast, GPs from the Netherlands and the UK felt they were already familiar with the content of the introduction section of intervention, which gave background to the problem of over-prescribing, and were initially less enthusiastic.

"“Some of the stuff I think is quite basic and I think it is more just using it as a teaching aid for medical students or GP registrars or even triage nurses. But normal GPs should hopefully have a lot of that basic skill already.” (British, GP1)."

However GPs often found later components, such as those describing techniques to help change their prescribing, were new to them or helped to reinforce their knowledge.

"“When I look at the communication training it makes me think… actually I already know these things, however I think that… a number of things… in particular drawing out the [patient] expectations and doing a good physical examination, which often I am tempted to just do quickly under the clothes to check their lungs, but doing it properly will reassure people. Yes, I think this is quite good… to do this again… to cross the T’s.” (Dutch, GP4)."

"“I think that’s quite a good illustration about how to specifically, just a revision, about how to specifically address patients concerns, because they… so often, it’s so important… it’s quite a good way of thinking about you know, a way to reinforce how you need to ask about those symptoms.” (British, GP4)."

#### Attractiveness and feasibility of the content of the interventions

GPs across all countries felt it was crucial that the training, whilst being relevant, should also provide specific benefit for them in their daily clinical practice. Whilst some felt that offering new knowledge was enough, most wanted to see clear advantages that would be obtained by following the intervention. Many GPs liked the idea of being provided with additional equipment in the form of the CRP test and the patient booklet and felt that receiving these would help them to decrease their inappropriate prescribing.

"“The purpose is going to be good but you’ve got to capture people who get 50 emails a day minimum and have to fit lots of education in, so GPs have to see straight away what they are getting out of the intervention for their daily practice.” (British, GP3)."

"“Indeed a CRP test could be reassuring with a borderline case… normally you would do on a Friday what you would never do on a Thursday. Then you prefer certainty over uncertainty which is a shame. Research shows that on Fridays more antibiotics are prescribed. However, I am also guilty of that and I know it… And this would surely help, so you can say no since the CRP is only 15. If it doesn’t get better, you can come back on Monday. This gives you the confidence to [make the decision]. I see this as very valuable.” (Dutch, GP1)."

As well as providing benefit to themselves, GPs sought reassurance that the training offered by the intervention could prevent harm to their patients whilst providing alternative management strategies that still maintained patient satisfaction. Again GPs felt that the additional equipment provided in the intervention would be acceptable for their patients. The CRP test was thought to be particularly helpful as it would give patients “evidence” on the seriousness of their condition; regardless of whether or not the test indicated a need for antibiotics.

"“I think that the act of carrying out a test and being able to give them a quantitative result is very much liked by patients. Patient satisfaction is generally higher if you can give them something that demonstrates that it is a viral infection and supports not having to use antibiotics.” (Spanish, GP2)."

"“I wonder if [the CRP test] would be a good tool to persuade patients who insist on antibiotics, who do not need antibiotics, and you go ‘look, you’ve had your blood test’. It’s like someone having an x-ray right in front of you, you can say ‘look your bone is not broken, go home’.” (British, GP5)."

Whilst all GPs felt that the intervention topic was relevant to their practice and felt the training would be beneficial for them to follow, some anticipated specific barriers in implementing the intervention which reflected the contexts in which they worked.

Some GPs reported that the consultation style and length portrayed in the communication training videos did not match their typical consultations in practice. One example of this was a GP from Poland who felt there were cultural differences in how patients and GPs communicated with one another in consultations between countries.

"“I think it is a copy of a British programme and a local specification should be taken into account. By local specification I mean both the length of time devoted to a patient and the way in which the doctor and the patient communicate with each other. I generally have no experience of such inquisitive patients…This is a wrong assumption that you can talk with every patient in this way. Most Polish patients expect the doctor to make a decision. They don’t expect to make a decision themselves or to be educated during a consultation. It is more about the doctor who has to pass information on to a patient. It is the doctor’s role to tell a patient why he is not going to prescribe an antibiotic.” (Polish, GP1)."

GPs in the Netherlands and Belgium also reported that they would feel they were patronizing patients if they asked them to sum up what they had learned at the end of the consultation.

"“I don’t think that in Belgium it is customary to ask the patient to summarise their account. I think many patients will find that strange (…) I think that we normally say ‘Is everything clear? Are there any questions?’ but not asking something like ‘what have we learnt today?’” (Belgian, GP2)."

GPs from Spain and Poland reported that they experienced problems when patients had already obtained antibiotics over the counter prior to a consultation, which they reported as a common concern for them.

"“I don’t know about other countries but patients come to get antibiotics which they have already bought in the pharmacy, so you’re under pressure to prescribe that antibiotic that they have been using… maybe we should have more information [on this]…” (Spanish, GP2)."

Belgian GPs highlighted the fact that reducing antibiotic prescriptions would lead to fewer consultations, which would result in less income for a practice in their fee for service system.

"“It is of course uncertain whether in Belgium one will be positive about this, that there will be fewer consultations. This is a system where people are paid on performance and [fewer consultations] is not something we are looking forward to.” (Belgian, GP4)."

Lastly, GPs from Belgium and Poland felt that information contained in the intervention about certain patient behaviours was incorrect for their patients. They felt their patients consulted more quickly than suggested in the intervention because of the need for a “sick note” to take time off work. GPs in these countries reported that patients would usually consult after having symptoms for one or two days rather than illnesses of over a week.

"“I think that our reality is a little bit different, because it says that patients consult a doctor seven to ten days after the initial symptoms start and I think that they do it a bit earlier. I think that they come to us on the third day…they don’t wait the whole week or a week and a half, because it is too long for them.” (Polish, GP2)."

Whilst the issue of context was mainly related to implementation, GPs also mentioned the relevance of context when looking at the evidence base of the intervention. Although GPs agreed that evidence was crucial to support guidance and interventions, many stressed that they would prefer evidence to be based on research in their own countries or to be provided with familiar sources, for example national guidelines for their country.

"“[Reading from INTRO webpage] ‘…a study in the US.’ Here we need to be cautious and make sure it can be extrapolated to our system. It also needs to be checked, whether there have been any studies done in Belgium, France or the Netherlands, i.e. that there are no European equivalent studies.” (Belgian, GP1)."

"“Unfortunately these studies are foreign. It is needless to say that our Polish market is different from the western one. I am not sure if the research that is conducted in general practice in Great Britain will have any meaning for Polish doctors.” (Polish, GP1)."

## Discussion

### Main findings

This was the first qualitative pilot study to explore GPs’ views on a specific intervention to reduce antibiotic prescribing for use in multiple countries. GPs in all five countries reported the view that the intervention addressed an important topic, was broadly acceptable and feasible to use, and would be a useful tool to help improve clinical practice. However, GPs in the different countries identified aspects of the intervention that did not reflect their national culture or healthcare system. Examples include differences in communication style, consultation length, and stage of illness in which patients typically consult.

GPs from Poland and Spain were concerned about the challenges that arose when patients were able to go directly to the pharmacy to receive antibiotics. Whilst illegal, the sale of antibiotics over the counter continues in Spain and it is possible to buy antibiotics in community pharmacies prior to obtaining a prescription [20,21]. GPs felt that by not addressing such issues the intervention did not provide them with the tools to overcome these difficulties. Polish and Belgian GPs explained that patients often present early on in the course of their illness due to the need for a sickness certificate. Presenting with diffuse symptoms may influence CRP results and affect management as patients may present with worse symptoms at day 1 than if they presented on day 7 of their illness. This has been recognised in previous research that has identified that the opportunity for self-certification may ameliorate the need for consultations [22]. GPs from Belgium reported mixed feelings about the implications of the intervention for their practice where a reduction in consultations in a fee for service system will result in reduced income [23].

### Study limitations

Participation in this study was voluntary and therefore the sample may represent those GPs who were more interested in the topic of antibiotic prescribing or in research in general. Self-report data are always vulnerable to participants giving socially acceptable answers. When presented with the study GPs were told that their views were important for helping to enhance the intervention for future use. They were particularly encouraged to talk about aspects they did not like about the intervention and interviewers made it clear they did not have personal involvement in developing the intervention.

### General recommendations for similar studies

Pilot studies offer the opportunity to test interventions and identify potential problems during development which may negatively affect implementation. Using qualitative methods to explore GPs’ views of the INTRO intervention and its influence on their practice, in all relevant contexts, helped us to identify relevant, local contextual barriers to behaviour change. The use of qualitative methods to identify potential problems with interventions, prior to implementation, is likely to help in identifying any aspects which clinicians may disengage with, at a time when the intervention can still be amended, and fits with recommendations to incorporate qualitative work into intervention development [11,12].

It is important for those who develop interventions for multiple contexts to ensure that the content covers a diverse range of situations relevant to all the target countries (for example a patient consulting at different stages of their illness) and provides alternative techniques to work towards changing practice (for example giving GPs several examples of how to use the same communication skills using different phrases). A suitable approach may be to create a core intervention and tailor aspects where needed to acknowledge differences in the delivery of health care between contexts.

In the present study, the content and phrasing of information was somewhat tailored for different countries. It is important to maximise the use of local information and primary care guidance to support main messages and to provide country-specific examples for GPs. Such tailoring will likely lessen the possibility of GPs disengaging with interventions or believing that they are not possible in their own practice and may ultimately help to increase an intervention’s effectiveness.

The effectiveness of the INTRO intervention at changing GP prescribing of antibiotics for acute cough is being tested in an RCT [Trial number: ISRCTN99871214]. As trials of interventions should be accompanied by qualitative investigations to help understand the ‘active ingredients’ of the intervention and to help interpret the results, we plan to undertake further qualitative work to assess how the final version of the INTRO intervention was implemented in practice by doing interviews with patients and additional interviews with GPs after participating in the trial.

## Conclusions

This study found that the INTRO intervention, which aims to support GPs in implementing evidence-based antibiotic prescribing behaviour for acute cough, was viewed as acceptable and feasible for implementation by GPs in multiple European countries. However, GPs identified ways in which the intervention could be modified to better reflect the local primary care context. Incorporating local information and guidance into interventions, and offering a choice of skills that GPs can implement as they feel suitable in their own context, are likely to improve implementation of such interventions.

## Abbreviations

GP: General Practitioner; INTRO: INternet TRaining for Oral antibiotic use; CRP: C-Reactive Protein; GRACE: Genomics to combat Resistance against Antibiotics in Community-acquired LRTI in Europe; LRTI: Lower Respiratory Tract Infection.

## Competing interests

The authors declare that they have no competing interests.

## Authors’ contributions

SA, STC, ED, LY, JC, NF, SC, TV, HG and PL were involved in discussions that led to the original idea of the research. SA, STC, PFV, JK and CL collected the data. SA, STC and ED were involved in analysing the data. SA and STC were responsible for writing the first draft of the paper. This study adheres to the RATS guidelines on qualitative research. All authors read and approved the final manuscript.

## Sources of support

This study was funded by the European Commission. In Flanders (Belgium) this work was supported by the Research Foundation – Flanders (G.0274.08 N).

## Pre-publication history

The pre-publication history for this paper can be accessed here:

http://www.biomedcentral.com/1471-2296/13/101/prepub
